# P-2355. Etiology and symptoms of respiratory tract infections among nursing home residents: Results from multiplex respiratory pathogen testing in the Nursing Home Public Health Response Network, February – April, 2024, United States

**DOI:** 10.1093/ofid/ofae631.2506

**Published:** 2025-01-29

**Authors:** Alfonso C Hernandez-Romieu, Tiffany G Harris, Sujan Reddy, Yasin Abul, David Canaday, Christopher J Crnich, Scott Fridkin, Sammantha Fuller, Jon P Furuno, Stefan Gravenstein, Steven Handler, Lindsay LeClair, Jennifer Meddings, Jennifer K Meece, Lona Mody, David A Nace, Paulina Rebolledo, Jennifer L Harcourt, Amanda B Payne, Rachel Slayton, Majerlee Reeves, Morgan Katz, Hannah L Kirking

**Affiliations:** CDC, Atlanta , GA; Abt International, Rockville, Maryland; CDC, Atlanta , GA; Brown University, Providence, Rhode Island; VA Northeast Ohio Healthcare System, Cleveland, Ohio; University of Wisconsin School of Medicine and Public Health, Madison, WI; Georgia Emerging Infections Program, Decatur, GA; Emory University School of Medicine, Atlanta, GA, Atlanta, Georgia; Abt International, Rockville, Maryland; Oregon State University, Portland, Oregon; Brown University, Providence, Rhode Island; University of Pittsburgh, Pittsburgh, Pennsylvania; Abt International, Rockville, Maryland; University of Michigan and the Ann Arbor VA Healthcare System, Ann Arbor, MI; Marshfield Clinic Research Institute, Marshfield, Wisconsin; University of Michigan, Ann Arbor, Michigan; University of Pittsburgh, Pittsburgh, Pennsylvania; Emory University School of Medicine, Emory University Rollins School of Public Health, Atlanta, GA; Division of Viral Diseases, Centers for Disease Control and Prevention (CDC), Atlanta, Georgia; CDC, Atlanta , GA; Centers for Disease Control and Prevention, Atlanta, GA; CDC, Atlanta , GA; Johns Hopkins, Stevenson, MD; Coronavirus and Other Respiratory Viruses Division, National Center for Immunization and Respiratory Diseases, CDC, Atlanta, GA

## Abstract

**Background:**

Respiratory tract infections (RTI) cause high morbidity and mortality among nursing home (NH) residents, yet their etiology and frequency remain understudied. We implemented multiplex respiratory panel (MRP) testing to describe RTI epidemiology in NH residents to inform efforts to reduce respiratory virus transmission in NH.

Figure 1

**Figure:**
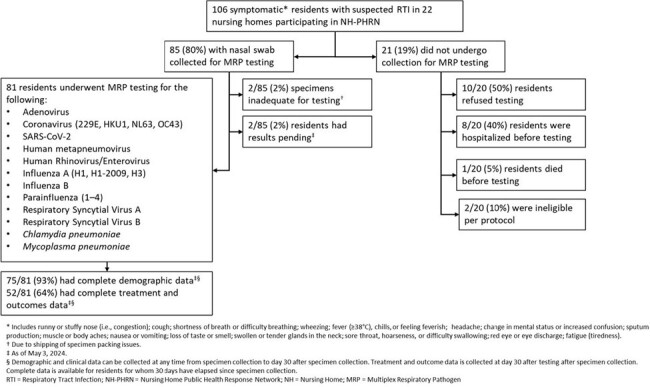

Flow diagram and multiplex respiratory panel testing results of SNF residents in NH-PHRN, February–April, 2024.

**Methods:**

The Nursing Home Public Health Response Network (8 academic sites and 22 affiliated NHs) is conducting RTI surveillance with MRP testing. Staff collect nasal swab (NS) specimens and clinical data from symptomatic residents with suspected RTI. Specimens are collected within 72 hours of symptom onset and tested with the Roche ePlex Respiratory Pathogen Panel 2 (17 viral and bacterial pathogens, Figure 1) in a central laboratory. We describe MRP results, symptoms, and characteristics of residents tested during February–April 2024. We compared symptoms between NH residents with and without viral detection on MRP with Chi-Squared and Fisher Exact tests.

Figure 2

**Figure:**
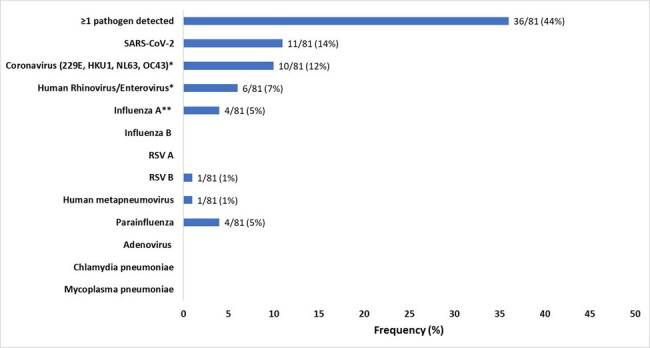

Multiplex respiratory pathogen testing results among 81 enrolled residents in 19 nursing homes in NH-PHRN, February – April 2024, United States. *One resident tested positive for both Human Rhinovirus/Enterovirus and Coronavirus. **Influenza A subtype was H3 for all four specimens.

**Results:**

Of 106 symptomatic residents with suspected RTI, 81 (72%) were tested (Figure 1). MRP detected ≥1 virus in 36/81 (44%) specimens; SARS-CoV-2 was the most common (14%), followed by seasonal coronavirus (12%) (Figure 2). Among residents tested, cough (78%), runny nose (43%), and sore throat (31%) were the most common symptoms. Sore throat was more common among residents with viral detection (53% vs. 13%; *P*=0.0001) (Figure 3). Of 75 tested residents with demographic and clinical data, median age was 80 (range 53–90), 59% were female, 19% were Black, 68% were long stay ( >100 days), and 99% had received a COVID-19 vaccine. Among 52 tested residents with treatment and outcome data, 14 (27%) received antibacterials, 11 (21%) were hospitalized, and 3 (6%) died. Of 25/52 residents with viral detection on MRP and complete treatment and outcome data, 4 (16%) received antibacterials, 6 (24%) were hospitalized (SARS-CoV-2 = 3, seasonal coronavirus = 2, Influenza A = 1) and none died.

Figure 3

**Figure:**
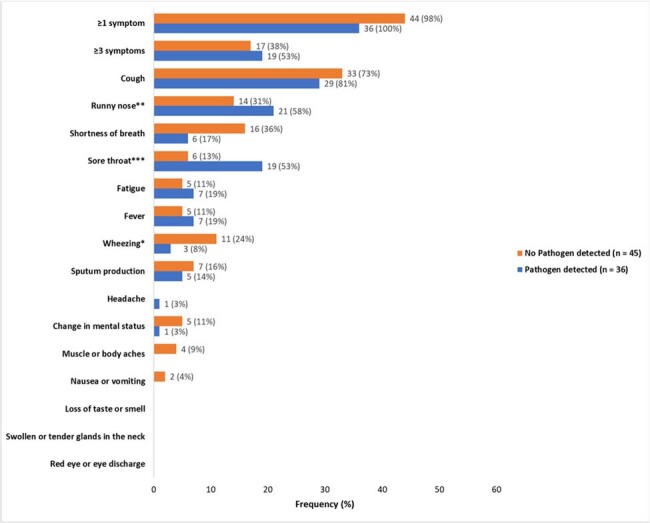

Frequency and percentage of symptoms and other clinical characteristics among 81 residents who underwent multiplex respiratory pathogen specimen collection in 19 nursing homes in NH-PHRN, February – April 2024, United States. We used Chi-Square and Fisher Exact tests to compare the type and frequency of symptoms among residents who had multiplex tests with and without pathogens detected. One asymptomatic resident who had no pathogen detected was tested due to leukocytosis. *Comparison P=0.04. **Comparison P=0.01. ***Comparison P=0.0001.

**Conclusion:**

MRP testing identified a virus in almost half of tested NH residents with suspected RTI at the end of the viral respiratory season. Ongoing surveillance will help describe the burden and clinical relevance of viral respiratory pathogens in NH residents and identify areas of improvement for respiratory virus infection prevention.

**Disclosures:**

Yasin Abul, MD, Moderna: Grant/Research Support|Moderna, Abt, CDC: Grant/Research Support David Canaday, MD, Moderna: Grant/Research Support|Pfizer: Grant/Research Support Jon P. Furuno, PhD, Merck & Co., Inc: Grant/Research Support Stefan Gravenstein, MD, MPH, CDC: Advisor/Consultant|CDC: Grant/Research Support|Genentech: Advisor/Consultant|Genentech: Grant/Research Support|Genentech: Honoraria|GlaxoSmithKline: Advisor/Consultant|GlaxoSmithKline: Grant/Research Support|GlaxoSmithKline: Honoraria|Janssen: Advisor/Consultant|Janssen: Grant/Research Support|Janssen: Honoraria|Moderna: Advisor/Consultant|Moderna: Grant/Research Support|Moderna: Honoraria|NIH: Grant/Research Support|Pfizer: Advisor/Consultant|Pfizer: Grant/Research Support|Pfizer: Honoraria|Sanofi: Advisor/Consultant|Sanofi: Grant/Research Support|Sanofi: Honoraria|Seqirus: Advisor/Consultant Lona Mody, MD, MS, Nanovibronix: Grant/Research Support Morgan Katz, MD, MHS, Ageless Innovation: Advisor/Consultant

